# The Need of Slanted Side Holes for Venous Cannulae

**DOI:** 10.1155/2012/854938

**Published:** 2012-01-12

**Authors:** Joong Yull Park

**Affiliations:** School of Mechanical Engineering, College of Engineering, Chung-Ang University, Seoul 156-756, Republic of Korea

## Abstract

Well-designed cannulae must allow good flow rate and minimize nonphysiologic load. Venous cannulae generally have side holes to prevent the rupture of blood vessel during perfusion. Optimizing side hole angle will yield more efficient and safe venous cannulae. A numerical modeling was used to study the effect of the angle (0°–45°) and number (0–12) of side holes on the performance of cannulae. By only slanting the side holes, it increases the flow rate up to 6% (in our models). In addition, it was found that increasing the number of side holes reduces the shear rate up to 12% (in our models). A new parameter called “penetration depth” was introduced to describe the interfering effect of stream jets from side holes, and the result showed that the 45°-slanted side holes caused minimum interfering for the flow in cannula. Our quantitative hemodynamic analysis study provides important guidelines for venous cannulae design.

## 1. Introduction

Cannula is one of the most primary tools for cardiac surgery, especially when the extracorporeal blood circulatory device is expected. A special care should be given in design of venous cannulae because, as opposed to arterial cannulae, they generally have side holes to improve hydraulic performance in suction of blood. This is particularly important in consideration of (1) that minimizing buffer volume for venous reservoir is required for better clinical outcome and (2) that a low central venous pressure limits an active control of blood flow rate for extracorporeal devices. Considering that the improvement of venous cannulae means a more stable and higher flow under lower venous pressure condition, the optimization of the side holes parameters (size, angle, position, arrangement, shape, etc.) is to be a practical target for improving the efficacy and safety of venous cannulae. In practical medical/animal test cases, doctors frequently suffers from the reduced intake volume to the extracorporeal system due to unstable blood suction through the venous cannulae; here, it should be noted that the side holes contribute to prevention of rupture of blood vessel (obstruction of cannula) during perfusion [1]. For the evaluation of performance of cannulae, the “M-number” (or “catheter number”) has been widely used [2]; the M-number is a single dimensionless number that is based on a Reynolds friction factor correlation and describes the pressure-flow relationship in a cannula. However, it only shows the bulk performance of cannulae and does not characterize other hemodynamic local phenomena such as vortices, flow stagnation/separation, and mechanical stress. Without deep understanding and close observation on the local flow in cannula, it is hardly possible to design and evaluate the cannulae appropriately.

From that point of view, computational fluid dynamics (CFD) models have many beneficial features such as visualization of local phenomenon, solutions for all physics parameters, low cost, and fast, to name a few. Grigioni et al. proposed a full-scale computational model for a cannula with side holes to predict the mechanical blood trauma and emphasized that a small shear stress can be a possible clinical problem [3, 4]. Also Park et al. examined the effect of the number and position of side holes on flow rate and shear stress using CFD and proposed a appropriate design of venous cannula with staggered array of side holes in view point of flow rate and shear rate [5]. However, none of previous studies have shown the effect of the slant angle of side holes in venous cannulae, while the angle of cannula side holes should be included as a primary parameter for the design and the study on its effects on hemodynamics such as local flow pattern, overall flow rate, and shear stress.

Mechanical stress and chemical agonists (e.g., platelet agonists that alter platelet shape and aggregation properties) can activate platelets and induce the release of additional platelet agonists, such as adenosine diphosphate (ADP) and thromboxane A2 (TxA2) [6]. Activated platelets, while important for wound healing, tend to stick to foreign surfaces and can cause significant problems for cannulae, valves, stents, oxygenators, tubes, and other artificial circulatory devices. Therefore, mechanical stress should be reduced as much as possible, and close observation is needed even at small shear rate (SR) values because the formation of rigid microaggregates can occur to low ADP concentrations (0.25–2.0 mmol) [3]. In addition, cumulative effects of blood cell damage can lead to clinical problems [3, 7, 8]. Thus, the results of our study of SR (Figure 5(b)) can guide the use and design of cannulae. These results show that increasing the number of side holes generally reduces the mechanical load placed on blood cells. Thus, in no side hole (NSH) cannulae, all flow proceeds from tip to outlet and all blood cells are exposed to a certain amount of mechanical load. As the number of side holes increases, a greater amount of blood joins the main stream from side holes, resulting in a reduction of exposure to mechanical load. Thus, increasing the number of side holes reduces the mean SR.

Knowing that the primary goals of cannula design is to achieve higher flow rates with lower shear stress to blood cells, here I report the effect of the angle of venous cannula side holes on flow rate, most primarily, by use of three-dimensional (3D) simulations and also studied flow patterns and shear rate distributions. Four different side hole angles (0°, 15°, 30°, 45°) and four different numbers of side holes (0, 4, 8, 12) were selected to investigate the effect of venous cannula design on function. Based on my previous study that shows a slight better performance found in staggered array of side holes [5], I adopted the staggered array for this study. For understanding the reason of the angle effect, I also proposed a new parameter, named “penetration depth (PD),” that explains a flow-mixing relationship between the main stream and the other streams from side holes. I formed a hypothesis that the smoother mixing pattern (lower PD) should be guaranteed for a better hemodynamic performance (higher flow rate and lower mechanical load) of venous cannula. Our results provide insight into the consequences of cannula design on mechanical stresses to blood cells and provide guidance for the future design of venous cannulae. Most practically, I claim that all the side holes should have a slanted feature for a better performance in clinical cases such extracorporeal circulatory system operation. 

## 2. Materials and Methods

### 2.1. Cannula Geometry and Computational Models

**Figure 1:**
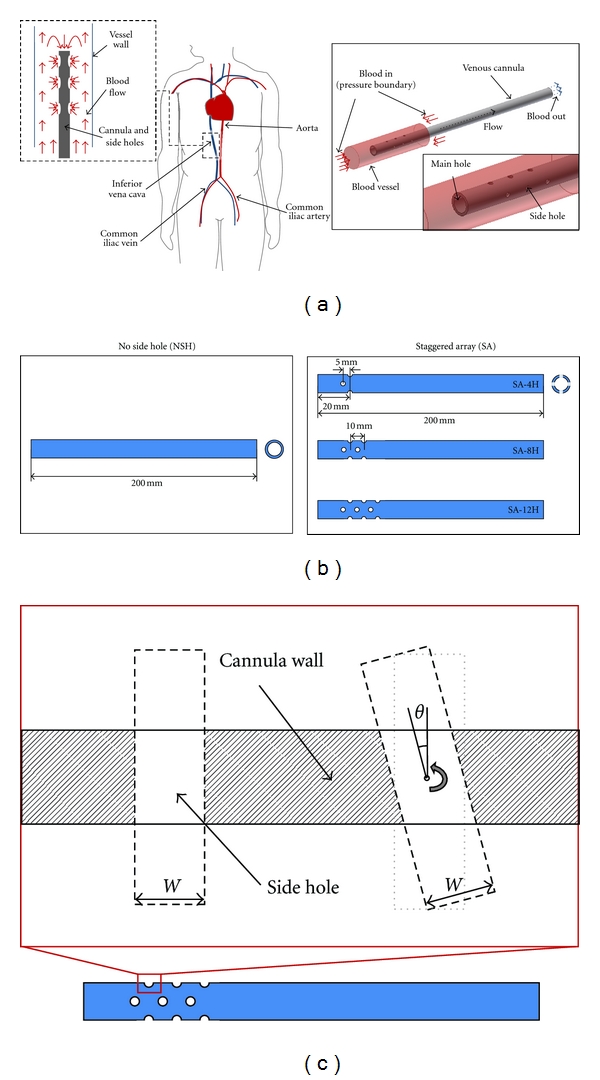
(a) A venous cannula inserted in the inferior vena cava was calculated (left). A computational domain was constructed in 3D (right). (b) Illustrations of cannula: NSH and SA models. (c) Cross-sectional view of side holes; left: vertically upright (0°) side hole; middle: inclined side hole of angel **θ** created by rotating the upright side hole geometry; right: inclined side hole of angel **θ** created by distorting the upright side hole geometry. All illustrations are not to scale except the 3D computational domain in (a).

Table 1 shows the geometrical parameters of the venous cannula used in our modeling. It was assumed that the cannula (inner diameter: 7 mm) was concentrically placed in a blood vessel of 20 mm diameter, similar to the size of the iliac vena cava [9] (Figure 1(a)). The models include no side hole model (NSH) and staggered array models (SA) [5] with different numbers (4, 8, and 12) and angles (0°, 15°, 30°, and 45°) of side holes (Figure 1(b)). The angle (**θ**) represents the rotation of the hole from its original vertical position (Figure 1(c)), with the hole diameter (2 mm) kept constant. Each model was named as “number of side holes-angle of side holes” (e.g., 12H-30°) and considered 13 different cannula designs.

**Figure 2:**
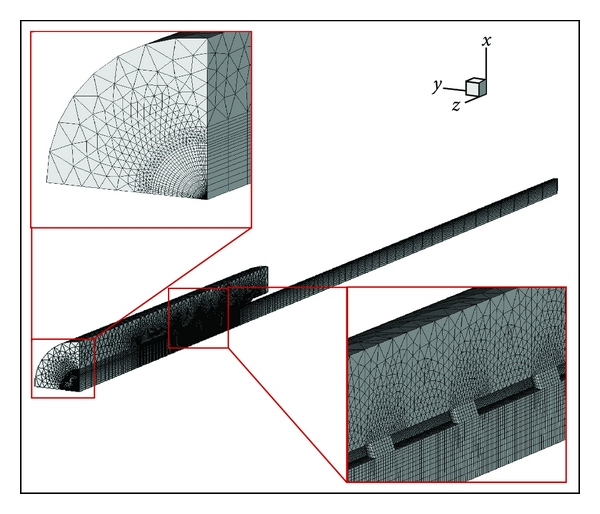
Grid system for model 12H-30° cannula. Insets show detailed views of the local mesh; pyramid grids are used for blood vessel part and structured grids for cannula. Also this figure shows that the 0.2 mm grid spacing is used near the side holes while a gradual increased spacing was applied to the distal area.

A finite volume method (FVM) model was created using a commercial program, FLUENT 6 (Fluent Inc.). In view of the cross-sectional symmetry of our cannula models, it was only necessary to simulate one-fourth of the geometry (Figure 2); a structured grid system was used for the inner cannula and pyramid/prismatic grids were used for the outer cannula region. I used the 60,000 grid counts for 12H models; typical grid spacing was about 0.2 mm near the side holes (see insets, Figure 2). It was considered a model to have converged on the result when the residuals of momentum and continuity equations reached 10^−12^. 

Flow was assumed to be laminar and steady, and blood (density of 1060 kg/m^3^,dynamic viscosity of 0.0035 kg/m·s) was assumed to be a homogeneous, incompressible Newtonian fluid. The Navier-Stokes flow motion equations and the continuity equation were used for modeling. Gravitational effects were not considered. The pressure boundary conditions were set at the proximal/distal inlets of the blood vessel and at the outlet of the cannula (right, Figure 1(a)). The pressure at the inlet was 10 mmHg and the pressure at the outlet was 0 mmHg, making the operative pressure difference (Δ*P*) 10 mmHg. This choice of Δ*P* was based on the practical reasons that the most favorable condition is when natural drainage volume is sufficient for the operation of extracorporeal circulatory system, and that the average venous pressure can be assumed to be 10 mmHg; it should be noted that the active drainage by operation of a circulatory pump system may cause higher pressure difference. Symmetric conditions were imposed to the *xy*-plane and the *yz*-plane (Figure 2). No slip boundary conditions were imposed at the blood vessel wall and at the internal and external walls of the cannula.

**Figure 3:**
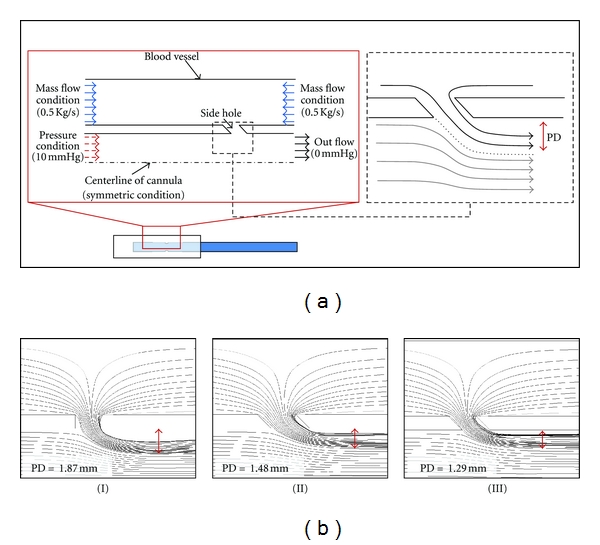
2D simulation results. (a) 2D computational geometry was constructed using the same dimensions from 3D model. However, to show clearly the effect of PD, different boundary conditions was applied; same pressure boundary were applied to the cannula inlet, and mass flow boundary was applied to the inlets from the blood vessel. (b) Therefore, the flow through the main inlet of cannula tip is dependent on PD value. By changing the angle to 45°, more amount of flow can be transferred to the main stream while the same flow rate is supplied through the side holes because of the application of mass flow inlet condition at the vessel part. Note that “I, II, and III” are the corresponding notations used in Figure 1(c).

As a supportive analysis, the flow distribution within the 2D cannula models was first visualized to evaluate the effect of side hole angle (Figure 3(a)). An axisymmetric model was used and the dimensions were same as 3D models. A fixed flow rate was applied to the outer inlets, while the pressure boundary was applied to the inlet of cannula.

### 2.2. Shear Rate

Apart from the flow rate, shear rate (SR), a scalar quantity, is another important hemodynamic factor that must be considered in the design and evaluation of medical devices through which blood flows. SR represents the rate of change of velocity and is equivalent to the first derivative of strain. In this paper, mean SR is a volume-averaged value of shear rates in the inner cannula space. I did not try to construct a very dense grid system to determine precise peak SR values. Mean SR appeared to be more meaningful, since the duration of maximum SR was observed only near the edge of side holes.

### 2.3. Penetration Depth

The angle of side holes may affect the direction of flow through the side holes. This flow can be described as “penetrating” the main stream, and thus disturbs the stream in the cannula. Therefore, apart from flow rate and SR, it was proposed a supportive parameter, “penetration depth (PD).” The PD is a penetrating distance of the stream coming through a side hole. Higher PD values mean that the main stream get disturbed greater by the flow streams from side holes. Therefore, the cannula design having lower PD values should be pursued.

## 3. Results and Discussion

In 2D cannula models, it was clearly showed that the angle of cannula side holes has a significant effect to the flow pattern in cannula. Corresponding to each geometry patterns (I, II, and III) shown in Figure 1(c), PD was 1.87, 1.48, and 1.29 mm (Figure 3(b)), and the flow rate was 2.83, 3.2, and 3.49 kg/s (note that this is 2D simulation). Here it was thus found that the geometry pattern II was not efficient as III and dropped in 3D modeling studies. Note that the bigger side hole size of pattern II (than that of pattern III) may allow a higher side hole flow rate; however, it should not be interpreted as the net flow of the pattern II cannula is also higher than that of the pattern III cannula.

**Figure 4:**
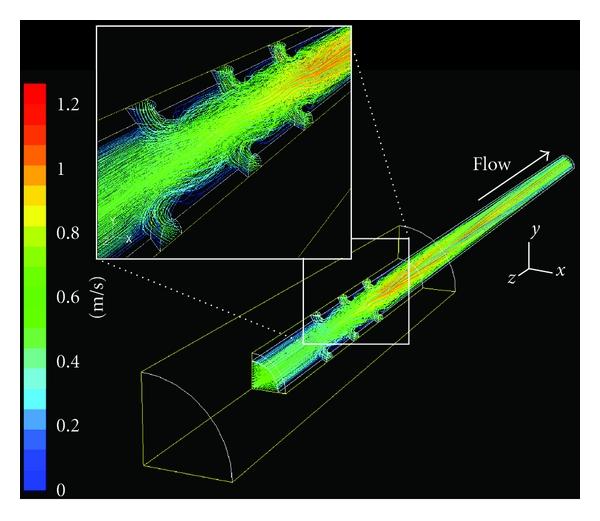
Streamlines of 12H-45°, with noticeable disturbance near the side holes. Inset shows detailed features of the entangled streamlines near the side holes.

**Figure 5:**
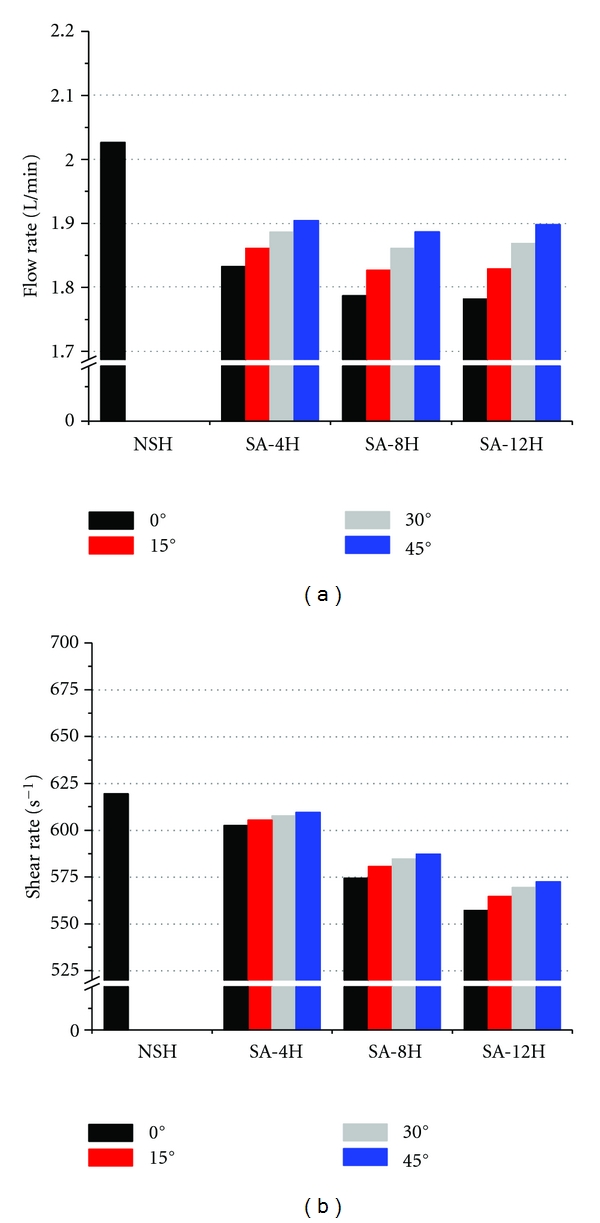
(a) Flow rates through different SA cannula models (inset shows the reduction of flow rate for each model compared to the NSH model). (b) Shear rates through different SA cannula models (inset shows the reduction of shear rate for each model compared to the NSH model).

In 3D cannula models, it was observed the disturbing pattern of flow.  Figure 4 shows the flow trajectory of a typical cannula (12H-45°); in this figure, the flow pattern outside the cannula was suppressed to clarify the flow distribution inside. The flow from side holes disturbed the main stream in the center of cannula, and thus the minimization of such disturbance should be achieved in new designs of venous cannulae. I evaluated the effect of the number and angle of side holes on cannula flow rates and SRs. As previously reported [5], the cannulae with no side holes (NSH) had the highest flow rate (2.03 L/min) (Figure 5). The flow rates of cannulae in which the side holes had no slant (0°) decreased as the number of side holes increased from 4 to 12 (blue dash-dot line, Figure 5(a)). However, by only slanting side holes, the flow rate increased about 3–5%. I also compared the SR of cannulae with different designs. NSH cannula had the highest SR (619 s^−1^) (Figure 5(b)). As the number of side holes increased to 12, the SR decreased by 11%. It is clear that the SR is strongly related to the amount of flow rate, however, the higher number of side holes are effective in reducing the shear stress. In addition, although wall shear stress is not dealt with in this research directly, it should be noted that the wall shear stress level is proportional to SR level in cannulae [5]. 

In agreement with the results of our previous studies [5, 10], it was found that flow rates in cannulae do not increase as the number of side holes increases from 4 to 12 (Figure 5(a)). It is very important to know that side holes can contribute to the safety of the cannulae, but have no “always-positive” effect on flow rate. A counterintuitive finding was that side holes possibly contribute to the perfusion of cannulae. Our side hole models had about 6–14% lower flow rate than the control (NSH) model (inset, Figure 5(a)). This may be because side holes disturb the flow pattern, with “tangled” streamlines appearing near the side holes (Figure 4). Cannula side holes appear to adversely affect the flow rate through a cannula [5]. However, in line with the results of previous studies [5, 10], the present study shows that a greater number (12 in our models) of side holes can recover the flow rate in some degree when the high side hole angle is presented (30° and 45° in our models).

**Figure 6:**
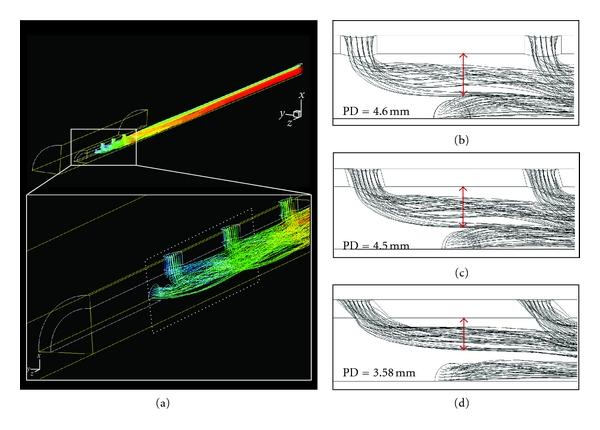
Effect of slanted side angle. (a) A typical model, 12H-45°, is presented to show the flow path distribution from the side holes. Inset is the zoomed feature near side holes. (b–d) PD reduces as the side hole angle increases, resulting in higher flow rate.

Our results showed that smoother flow pattern occurs as the angle of cannula side holes increases, resulting in higher flow rate (Figure 5(a)) and higher shear stress (Figure 5(b)). It is clear that the benefits of flow rate compensate the shear stress gained; however, a special care must be taken to minimize the shear rate. I claim that this can be achieved by slanting side hole. As the velocity contours of the 12H-45° models show (Figure 6(a)), the strength of flow from a side hole toward the center of the main stream (expressed as a PD) gets smaller from PD = 4.6 to 3.58 mm as the side hole angle increases from 0° to 15° and 45° (Figures 6(b)–6(d)). A deeper penetration of this flow will decrease the net flow rate through a cannula, and this is accompanied by a flow rate increase from 1.78 to 1.9 L/min. PD is inversely proportional to the side hole angle, resulting in an increased flow rate for more sharply angled side holes. This also applied to all of our other models with side holes. Thus, PD can be used to determine the effect of the side hole parameters on flow through a cannula.  Table 2 shows the PDs in each model.

With regard to flow rate, the best cannula design is the NSH model. However, since side holes allow for safer implementation of cannulae, cannulae with 4–12 side holes appear to be good alternatives. The SRs of the 12H-0° cannula (557.2 s^−1^) were the lowest. Ideal cannula design/use may depend on the unique requirements imposed by specific clinical situations. However, the 12H-45° cannula seems to have the best design for general use because it is associated with minor loss in flow rate and has moderately small SRs (Figure 5(a)).

Our study has several limitations. First, it was assumed a steady-state system with constant pressure boundary, concentrically poised cannula, and rigid blood vessel walls. I suggest that the especially the elasticity of the blood vessels be considered in future work because this can affect the rupture and closing of cannula side holes. Regardless, the general conclusions of our quantitative comparative study about the number and angle of cannula side holes remain valid.

## 4. Conclusion

In this study, 3D numerical analysis was used to investigate the effect of the angle of side holes. The primary target of our study is to improve the blood flow rate by slanting the side hole, and this can be additionally adapted with any other major improvement in cannula designs, and thus, I claim that all the side holes should be equipped at a hydrodynamically designed angle for better clinical outcomes. It cannot be too emphasized when considering that it can be a life-threatening problem for patents during medical operations. In particular, our study has two significant findings. First, when the side hole angle was 0°, a smaller number of side holes was associated with higher flow rate, while a larger number of side holes was associated with reduced mechanical SR. Second, when the side hole angle is 45°, a larger number of side holes allow a higher flow rate and greater reduction of SR. Therefore, there appear to be definite advantages of venous cannulae with a large number of side holes at an acute angel. I suggest that cannula design also depends on the clinical features of individual cases and should be based on a deeper understanding of cannula hemodynamics. Further study should be continued for improvement other parameters such as side hole shape and spacing. 

## Figures and Tables

**Table 1:** Geometrical dimensions of model cannulae and blood vessel.

Length	200 mm
Inner diameter	7 mm
Wall thickness	1 mm
Side hole diameter	2 mm
Side hole interval	10 mm
Array type	Staggered array
Angle of side holes	0°, 15°, 30° and 45°
Number of side holes	0 (no side holes), 4, 8 and 12
Blood vessel diameter	20 mm
Blood vessel length	80 mm

**Table 2:** Penetration depth in cannula models.

Cannula model	PD
4H-0°, −15°, −30°, and −45°	4.8, 4.62, 4.4 and 3.65 mm
8H-0°, −15°, −30°, and −45°	4.7, 4.6, 4.35 and 3.6 mm
12H-0°, −15°, −30°, and −45°	4.6, 4.5, 4.32 and 3.58 mm
